# Gene editing improves endoplasmic reticulum-mitochondrial contacts and unfolded protein response in Friedreich’s ataxia iPSC-derived neurons

**DOI:** 10.3389/fphar.2024.1323491

**Published:** 2024-02-14

**Authors:** Priyanka Mishra, Anusha Sivakumar, Avalon Johnson, Carla Pernaci, Anna S. Warden, Lilas Rony El-Hachem, Emily Hansen, Rafael A. Badell-Grau, Veenita Khare, Gabriela Ramirez, Sydney Gillette, Angelyn B. Solis, Peng Guo, Nicole Coufal, Stephanie Cherqui

**Affiliations:** ^1^ Department of Pediatrics, Division of Genetics, University of California, San Diego, San Diego, CA, United States; ^2^ Department of Pediatrics, University of California, San Diego, San Diego, CA, United States; ^3^ Sanford Consortium for Regenerative Medicine, La Jolla, CA, United States; ^4^ Department of Cellular and Molecular Medicine, University of California, San Diego, San Diego, CA, United States

**Keywords:** Friedreich’s ataxia, induced pluripotent stem cells, neurons, gene editing, neuronal apoptosis, calcium homeostasis, endoplasmic reticulum-mitochondrial contacts, unfolded protein response

## Abstract

Friedreich ataxia (FRDA) is a multisystemic, autosomal recessive disorder caused by homozygous GAA expansion mutation in the first intron of frataxin (*FXN*) gene. FXN is a mitochondrial protein critical for iron-sulfur cluster biosynthesis and deficiency impairs mitochondrial electron transport chain functions and iron homeostasis within the organelle. Currently, there is no effective treatment for FRDA. We have previously demonstrated that single infusion of wild-type hematopoietic stem and progenitor cells (HSPCs) resulted in prevention of neurologic and cardiac complications of FRDA in YG8R mice, and rescue was mediated by FXN transfer from tissue engrafted, HSPC-derived microglia/macrophages to diseased neurons/myocytes. For a future clinical translation, we developed an autologous stem cell transplantation approach using CRISPR/Cas9 for the excision of the GAA repeats in FRDA patients’ CD34^+^ HSPCs; this strategy leading to increased *FXN* expression and improved mitochondrial functions. The aim of the current study is to validate the efficiency and safety of our gene editing approach in a disease-relevant model. We generated a cohort of FRDA patient-derived iPSCs and isogenic lines that were gene edited with our CRISPR/Cas9 approach. iPSC derived FRDA neurons displayed characteristic apoptotic and mitochondrial phenotype of the disease, such as non-homogenous microtubule staining in neurites, increased caspase-3 expression, mitochondrial superoxide levels, mitochondrial fragmentation, and partial degradation of the cristae compared to healthy controls. These defects were fully prevented in the gene edited neurons. RNASeq analysis of FRDA and gene edited neurons demonstrated striking improvement in gene clusters associated with endoplasmic reticulum (ER) stress in the isogenic lines. Gene edited neurons demonstrated improved ER-calcium release, normalization of ER stress response gene, *XBP-1,* and significantly increased ER-mitochondrial contacts that are critical for functional homeostasis of both organelles, as compared to FRDA neurons. Ultrastructural analysis for these contact sites displayed severe ER structural damage in FRDA neurons, that was undetected in gene edited neurons. Taken together, these results represent a novel finding for disease pathogenesis showing dramatic ER structural damage in FRDA, validate the efficacy profile of our *FXN* gene editing approach in a disease relevant model, and support our approach as an effective strategy for therapeutic intervention for Friedreich’s ataxia.

## 1 Introduction

Friedreich’s ataxia (FRDA) is a progressive, lethal, multisystemic, autosomal recessive disorder, predominantly caused by homozygous GAA trinucleotide expansion mutation in intron 1 of the frataxin (*FXN*) gene. FRDA patients typically display 44 to 1700 GAA repeats in comparison to 5–30 repeats in healthy individuals (Pandolfo, 2009). This expansion mutation results in partial transcriptional repression of the gene, either through epigenetic modifications or heterochromatin formation (Punga and Buhler, 2010), and strong reduction in protein levels ranging from 5% to 35% in patients compared to healthy adults (Campuzano et al., 1997). FXN is a ubiquitously expressed mitochondrial protein that participates in iron-sulfur (Fe-S) cluster biosynthesis and thus, its deficiency is associated with compromised electron transport chain functions, redox imbalance and increased mitochondrial iron load (Llorens et al., 2019). The resulting toxic biochemical changes augment cell death and primarily affects mitochondria-rich tissues like the nervous system, skeletal muscle, and heart. Neurological symptoms such as progressive limb and gait ataxia, proprioception loss and dysarthria typically begins between 5 and 15 years of age (Harding, 1981) and affected individuals become wheelchair bound within 10–15 years of symptoms onset. The predominant cause of mortality in patients is hypertrophic cardiomyopathy (Payne and Wagner, 2012).

The neurological phenotype results from degenerating cerebellar neurons, sensory neurons in the dorsal root ganglion (DRG), dentate nucleus of the cerebellum, motor cortex and the corticospinal tracts (Koeppen and Mazurkiewicz, 2013; Harding et al., 2020). However, these cell types are generally inaccessible from patients for understanding disease pathogenesis, developing, and validating therapeutic approaches. Mouse models of FRDA such as the NSE-Cre or YG8 lines recapitulate some but not all overt neurodegenerative phenotypes seen in patients (Perdomini et al., 2013; Gerard et al., 2023). Thus, induced pluripotent stem cells (iPSC) derived from FRDA patients’ fibroblasts and other cells have gained traction in recent years. Indeed, several research groups have generated FRDA iPSC-derived neurons and these cells are morphologically unaffected, display delayed functional maturation, oxidative stress, decreased Fe-S containing proteins and reduced mitochondrial membrane potential (Hick et al., 2013; Soragni et al., 2014; Igoillo-Esteve et al., 2015; Codazzi et al., 2016), effectively recapitulating some of the critical phenotypes of the disease. Thus, these iPSC-derived neurons are potentially ideal candidates for translational studies of both pharmacological interventions and genetic manipulations.

We previously reported that a single infusion of wild-type mouse hematopoietic stem and progenitor cells (HSPC) into lethally irradiated YG8R FRDA mice prevented progression of both the neurologic and cardiac complications of the disease and tissue rescue was mediated by the tissue engraftment of HSPC-derived macrophages/microglia that transferred functional mitochondria to myocytes/neurons (Rocca et al., 2017). For an autologous translational approach, we utilized CRISPR (Clustered Regularly interspaced short-palindromic repeat) - Cas9 (CRISPR-associated 9) gene editing technology to excise the GAA trinucleotide expansion from intron 1 of *FXN* in FRDA patients’ CD34^+^ HSPCs. Following gene-editing these cells retained hematopoietic reconstitution potential, demonstrated physiological rescue of *FXN* expression and improved mitochondrial biomarkers (Rocca et al., 2020). However, clinical translation of these approaches requires robust and disease-relevant models to demonstrate efficacy and safety.

Here. We report on the efficacy of our CRISPR/Cas9 gene-editing approach utilizing a cohort of different FRDA iPSC-derived neurons, carrying varying lengths of GAA expansion with characteristic FRDA phenotypes. *FXN* gene editing improves neuronal and mitochondrial morphology, decreases cellular apoptosis, and displays distinct transcriptomic signatures representative of improved mitochondria-endoplasmic reticulum (ER) interactions. We also demonstrate that gene-edited neurons retain mitochondrial-ER contact sites for calcium homeostasis as well as augmented unfolded protein response, critical for functional control and communication between the organelles. Our results reveal novel gene signatures with potential implications in FRDA disease pathogenesis and demonstrate that our gene-editing approach represents an efficient therapeutic approach for FRDA in a disease-relevant model.

## 2 Materials and methods

### 2.1 Cell lines

In this study, we utilized the iPSC FRDA FF1 cell line generated from FA patient fibroblast GM03816 (Coriell Institute) (Hick et al., 2013), and isogenically corrected E35 line generated by replacing the long expansion by a shorter, healthy repeat number as healthy control (Codazzi et al., 2016). These cells were kindly provided by Dr. Joel Gottesfeld and Dr. Elizabeth Soragni (The Scripps Research Institute). The iPSC FRDA FF2 line (denoted as in graphs) was purchased from Coriell Institute (#GM23913). Patient lymphoblast cell lines (LCLs) #GM15850 and #GM16223 and healthy control #GM22264 were purchased from Coriell Institute (called 850, 223 and 264, respectively, henceforth). Both the above mentioned patient LCLs and FF2 iPSC lines were gene edited for *FXN* hyper expansion mutation with our optimized CRISPR/Cas9 protocol (Rocca et al., 2020) as delineated below. All cell lines were utilized for all studies unless specifically mentioned. “Control” represents 264 (unrelated control line) and E35 (isogenically corrected line of FF1); “FRDA” includes FF1, FF2, 223 and 850 lines while “Edited” includes FF2, 223 and 850 lines that were CRISPR/Cas9 gene edited for *FXN*.

### 2.2 iPSC generation

LCLs were reprogrammed via the Epi5 Episomal iPSC Reprogramming Kit (Life Tech Cat#: A15960). Cells were counted using trypan blue and 2 × 10^6^ LCLs were transferred to a 15 mL Falcon tube and centrifuged. The supernatant was completely aspirated, and cells were resuspended in Nucleofector Solution to prime cells for nucleofection. The cell suspension was combined with either pmax GFP Vector to evaluate nucleofection efficiency or Epi5 reprogramming vectors (Sox2, Oct4, Lin28, Klf4, L-myc) and EBNA to reprogram the cells to stem cells. After nucleofection, cell solution was added to pre-equilibrated media containing N2 (Thermo-Fisher) and B27 (Thermo-Fisher) additives and seeded at various densities on Matrigel-coated 6 well plates. iPSCs were then plated at low density to allow growth of individual colonies. iPSCs were maintained in mTESR1 plus medium (Stemcell Technologies).

CRISPR/Cas9 gene corrected iPSCs, present as mixed population of cells, were dissociated into single cells with 1 mL of Triple and plated at a density of 6k-8k cells/well on a 96 well plate. After the single cell colonies reached the desired size, colonies were isolated with Collagenase IV to individual wells of a 96 well plate and clones were then screened for presence and absence of gene editing with PCR. Selected clones were validated to have normal karyotype through DNA fingerprinting and then differentiated into NPCs. All cell lines were regularly verified to be free of *mycoplasma* contamination.

### 2.3 PCR for evaluation of the GAA repeat excision

Genomic DNA was extracted from iPSCs using QuickExtract (Lucigen, Middleton, W1, United States). PCR was carried out using 15–100 ng of gDNA for Gene Editing (GE) and Non-Gene Editing (NGE) respectively. Reactions were always performed separately but on gDNA derived from the same sample. Reactions included GoTaq Green Master Mix 2X (Promega, GmbH) and the following primers: GE fwd: 5′-GGT GTA GGA TTA AAT GGG AAT AA-3’; Rev: 5′ GGA TGC ACA GGA GCT TATT-3′, Non-Gene Editing NGE fwd: 5′- GGA CCT GGT GTG AGG ATT AAA-3’; rev: 5′-CTA ATA CAT GCG GCG TAC CA-3′ as previously described (Rocca et al., 2020). The following PCR settings were used: 95°C for 3min (95°C for 30s, 52°C (GE) and 56 °C (NGE) for 30s, 72 °C for 1min) x 35, 72 °C for 10min. PCR products were then run on a 1% agarose gel at 120V for 30min. GE and NGE bands were detected at 401 bp and 313 bp, respectively using a 1 Kb DNA ladder.

### 2.4 Neuronal progenitor generation

Neural progenitors were generated as previously described (Vadodaria et al., 2019). Briefly, iPSC colonies of approximately 50–100 cells in size were lifted off from the plate with collagenase diluted in DMEM F12 (GibcoCAT#11330–032). The colonies were then transferred onto a 6 well ultra-low attachment plates (Corning Ref#CLS3471) and placed on a shaker with gentle agitation for embryoid body formation in neural induction media (NIM; DMEM/F12 containing N2 and B27)) supplemented with Noggin (100 ng/mL), LDN193189 (100 nM) and SB431542 (10 µM). On Day 17, embryoid bodies were plated onto polyornithine and laminin coated 10 cm plates for rosette formation in NIM media supplemented with fibroblast growth factor 2 (20 ng/mL) and laminin (1 μm/mL). On Day 24, the rosettes were mechanically selected and enzymatically dissociated in Accutase (Chemicon). The resulting cell solution was subsequently plated onto polyornithine and laminin coated plates for neural progenitor cell expansion and maintained as a monolayer at high density.

### 2.5 iPSCs immunofluorescence

Control, FRDA and gene-edited iPSCs were fixed in 4% PFA for 20min and washed three times with TBS for 5min each. Coverslips were blocked using Normal Horse Serum (R37625 ThermoFisher) in TBS with 0.1% PBS-Triton-X100 (TBS++) for 60min and incubated with the following primary antibodies Oct4 (Cell-signaling Cat-2750s) and TRA1-60 (Invitrogen; MA1-023) overnight at 4°C. The following day, coverslips were washed three times in TBS for 5min each, followed by secondary antibodies incubation in TBS++ for 60min. After washing coverslips were mounted and imaged using a Keyence microscope with a ×10 objective.

### 2.6 DNA fingerprinting

Karyotyping was performed using 200 ng of DNA hybridized to Infinium CoreExome-24 arrays (Illumina) and stained per Illumina’s standard protocol. Copy Number Variation (CNV) calling was carried out in Nexus CN (version 7.5) and manually inspected, visualizing the B-allele frequencies (proportion of A and B alleles at each genotype) and log R ratios (ratio of observed to expected intensities) for each sample, as described (Panopoulos et al., 2017).

### 2.7 Neuron differentiation

Neuronal Precursor Cells (NPC) were maintained in NPC media, containing DMEM/F12 with Glutamax (Cat. #10565010, ThermoFisher Scientific, MA), 1X B27 Supplement, minus Vitamin A (Cat. #12587070, ThermoFisher Scientific, MA), 1X N-2 Supplement (100X) (Cat. #17502048, ThermoFisher Scientific, MA), Laminin (Cat. #A25607, ThermoFisher Scientific, MA) and FGF2 (Joint Protein Central, Korea).

Differentiation of NPCs to neurons was performed for varying differentiation times including 1, 2, 3, and 4 weeks with media change every 48hrs. Differentiation media contained DMEM/F12 with Glutamax (Cat. #10565010, ThermoFisher Scientific, MA), 1X B27 Supplement (Cat. #17502048, ThermoFisher Scientific, MA), 1X N-2 Supplement (100X) (Cat. #17502048, ThermoFisher Scientific, MA), Laminin (Cat. #A25607, ThermoFisher Scientific, MA), BDNF (Cat. #450–02-1mg, Peprotech, NJ, United States), GDNF (Cat. #450–10-1mg, Peprotech, NJ, United States), 1 mM dibutryl cyclicAMP (cAMP) (Cat. #1141, Tocris Bioscience, UK (and 200 nM ascorbic acid (Cat. #72132, StemCell Technologies, United States).

### 2.8 Magnetic labeling and cell isolation

One week differentiated neuronal cell suspensions containing approximately 3 × 10^6^ cells were first labeled with anti-PSA-NCAM antibody (catalog number: 130–097-859, Miltenyi Biotec) and subsequently, magnetic MicroBeads were employed. These cells were then introduced into an MS-column (Miltenyi Biotec) positioned within the magnetic field of a MACS Separator. The column underwent three washes with 0.5 mL of medium. PSA-NCAM positive cells, magnetically isolated during this process, remained within the column and were released as the positively selected cell fraction once the column was removed from the magnet. >94% of the cells were PSA-NCAM positive. This *in vitro* derived PSA-NCAM positive isolated cells were cultured on Matrigel-coated plates for further differentiation into neuronal lineage for immunocytochemistry or they were collected for RNA and protein isolation.

### 2.9 cDNA library preparation for mRNA sequencing

To perform the mRNA sequencing, total RNA was isolated using the RNeasy Tissue kit (Qiagen, Hilden, Germany, cat. 74104) according to the manufacturer’s instructions. RNA was assessed for quality using an Agilent Tapestation 4,200, and 50 ng of RNA with an RNA Integrity Number (RIN) greater than 8.0 were used to generate RNA sequencing libraries using the Illumina Stranded mRNA Prep (Illumina) following manufacturer’s instructions. The resulted libraries were multiplexed and sequenced with 100 base pairs (bp) Paired End reads (PE100) to a depth of approximately 25 million reads per sample on an Illumina NovaSeq 6,000. Samples were demultiplexed using bcl2fastq Conversion Software (Illumina).

### 2.10 mRNA seq data analysis

FASTQ files from sequencing experiments were mapped to hg38. RNA-seq files were mapped using the alignment tool STAR (v2.7.10b). Raw RNA-sequencing files were evaluated for quality control using FastQC (v0.12.0). Gene expression raw counts was quantified by HOMER’s ‘analyzeRepeats’ as previously described (Gerard et al., 2023). Differential gene expression was calculated using the HOMER command ‘getDiffExpression.pl’. Transcript per kilobase million (TPM) was quantified for all genes matching accession number to raw counts. Quantified read counts were normalized and analyzed using DESeq2 (v1.12.3) to determine differential expression between groups, within the R statistical computing environment (v4.2.2) (Love et al., 2014). Significantly differentially expressed genes (DEGs) were set at a threshold of padj n < 0.05 and log2 fold change >1. Genes with TPM <2 were removed from the analysis. Functional enrichment of pathways and Gene Ontology categories was performed using Metascape (Zhou et al., 2019). Significant enrichment was defined as a -log_10_ *p*-value >2 and a minimum of three genes per enrichment category. For upstream regulatory analysis (URA) of DEGs we used the commercial QIAGEN’s Ingenuity^®^ Pathway Analysis (IPA^®^, QIAGEN Redwood City). For URA, we used the Bonferroni method to correct the detection *p*-value and set P_ad j_< 0.05 as the significant threshold as the input method. Plots were generated using ggplot2 (v3.4.1) or Prism (v9.01, GraphPad, San Diego, CA).

### 2.11 Immunocytochemistry and blebbing analysis

Neurons were fixed in 4% paraformaldehyde (PFA) for 15 min and washed thrice with PBS. Permeabilization and blocking was performed with 3% bovine serum albumin (BSA, Sigma-Aldrich), 0.1% Triton X-100 (Sigma Aldrich) in PBS for 1 h at room temperature. The glass bottom Ibidi dishes were then incubated overnight at 4°C with primary antibodies (Anti-β-tubulin III: Mouse 1:300, ab78078 and Anti-Caspase 3 Antibody, active (cleaved) form: Rabbit 1:50, ab3623) diluted in solution containing 3% BSA. Next, PBS was used to wash the primary antibodies, followed by incubation with secondary antibodies (Alexa Fluor Dyes: 1:1000 dilution, Abcam) in a solution containing 3% BSA for 1 h at room temperature. Nuclei were visualized with DAPI (1:25,000, Life Technologies). Slides were mounted using ProLong Gold antifade reagent (Invitrogen) and images were acquired using Leica SP8 confocal microscope and Keyence microscope. Images were processed and analyzed with Image Pro-software. To quantify dendritic blebbing, dendrites were traced, and the number of blebs were automatically counted by ImagePro. The number of caspase positive cells were also automatically counted by ImagePro. Image analysis and quantification was performed by the trained experimenter blind to the conditions.

### 2.12 Electron microscopy (EM)

*Sample Preparation:* For EM sample preparation, we performed all steps on ice. After 2 weeks of differentiation, cells were fixed with 2% glutaraldehyde and 0.1M sodium cacodylate buffer (SC buffer) for 60 min. After fixation, the cells were washed five times for 3 min each in 0.1M SC buffer to remove excess fixative. The samples were then incubated for 30 min-1 h in a 1% OsO_4_ buffer. Fixed samples were dehydrated by immersion in a series of ethanol solutions with increasing concentrations: 20%, 50%, 70%, 90%, and finally 100%. Each dehydration step was carried out for 1 min. Following dehydration, the samples were incubated in 100% Durcupan for 1 h. After incubation, the cells were embedded in foil plates with wooden sticks. This embedded assembly was placed in an embedding oven for 36 h.

*Sectioning, imaging, and analysis:* 60 nm ultrathin sections were cut with diamond knife and mounted on 300 mesh grids. Grids were post stained with 2% of UA 5min and lead 1min. Images were captured on JEOL 1400 plus TEM at 80 KV with Gatan 4kx4k camera. Quantification of mitochondrial area and number were conducted in a blinded fashion. We used ImageJ software to count the number of mitochondria per cells following point-counting method (Bugger et al., 2009) and mean area with the freehand tool (Neikirk et al., 2023).

### 2.13 Western blot

Cell pellets from 2 weeks differentiated neurons were homogenized in RIPA Lysis Buffer (Cat. #89900, Invitrogen, MA) and 1X Halt Protease Inhibitor Cocktail (Cat. #87786, ThermoFisher Scientific, MA) and centrifuged at 10000 *g* for 15 min at 4°C. Protein concentrations were quantified with BCA Aassay (Cat. #23227, ThermoFisher Scientific, MA), as per manufacturer’s instructions. Equal quantities of proteins were separated on NuPage 4%–12% Bis-Tris Gel (Cat. #NP0321BOX) and transferred onto PVDF membrane (IB24001) with iBlot2 (ThermoFisher Scientific, MA). Membranes were blocked in 5% BSA, prepared in 1X TBS+0.1% Tween-20 (TBST) and incubated overnight with 1:1000 dilution of primary antibodies at 4°C: NDUFB8 and SDHB (Cat. #ab110413, Abcam UK) and FXN (Cat. #14147-1-AP, Proteintech Group Inc, United States). Membranes were washed TBST and incubated with anti-mouse secondary antibody conjugated with HRP (Cat. #A28177, Invitrogen, MA) and developed with Enhanced Chemiluminescent Substrate (Cat. #32106, ThermoFisher Scientific, MA) in Azure 600 imager. Β-Actin (Cat. #sc47778 HRP, Santa Cruz Biotechnology, CA) was used as loading control. Bands were quantified with GelQuant analysis software. All cell lines were utilized for protein expression studies and data generated from 2-3 biological repeats of differentiation of each line.

### 2.14 MitoSOX live cell imaging

Neurons differentiated for 2 weeks were stained with MitoSOX (Cat. #M36008, ThermoFisher Scientific, MA) at 100 nM concentration, MitoTracker Green (Cat. **#**9074S, CST) at 100 nM for 15mins, at 37°C in culture medium. Cells were washed with 1X PBS and counter stained with nuclear Hoechst. Cells were immediately imaged with Keyence Fluorescent Microscope and MitoSOX fluorescence intensity was quantified with ImagePro. Data is represented as relative fluorescence intensity of control cells.

### 2.15 Gene expression studies

Total RNA was extracted using RNeasy Mini Kit as per manufacturer protocol (Cat. #74104, Qiagen, Germany) from 2 weeks differentiated neurons. 500ng of RNA was converted to cDNA using iScript cDNA Synthesis Kit (Cat. #1708840, Bio-Rad, United States). All cell lines were utilized for gene expression studies and cDNA was generated from 2-3 biological repeats of differentiation of each line.

*RT-PCR for XBP-1 gene expression*: The Reaction assembly of the RT-qPCR consisted of 5 μL iTaq universal SYBR Green Supermix (Cat.#1725121, Bio-Rad, United States), 3 μL of 1:10 diluted cDNA and 1 μL forward and reverse primer (5 μM). The reaction was run on CFX96 thermocycler (Bio-Rad, United States) using the following conditions: 95°C (30 s); 40 cycles of 95°C (5 s) and 60°C (30 s); then 65°C (5 s); and 95°C (5 s). Gene expression was measured using the delta/delta CT method and normalized to glyceraldehyde 3-phosphate dehydrogenase (GAPDH) for total XBP1 (tXBP1). For the spliced (sXBP1) and unspliced (uXBP1), the gene expression was measured using the delta/delta CT method relative to total XBP1 (tXBP1) and normalized to glyceraldehyde 3-phosphate dehydrogenase (GAPDH). All primer sequences are shown in Supplementary Table S1.

ddPCR for FXN gene expression: Human FXN primer (Cat.#10031252, Bio-Rad, Hercules, United States) and TBP as reference gene (Cat.#10031255, Bio-Rad, Hercules, United States) were used for analyzing frataxin gene expression. Reaction setup consisted of 2 μL of cDNA, 1 μL of each primer, HindIII and 1x ddPCR supermixes for probes (no dUTP) (Bio-Rad, Hercules, United States of America). Droplets were generated with QX200 droplet generator and then transferred to a 96-well plate. PCR conditions and analysis were performed as described before (Rocca et al., 2020).

### 2.16 Calcium live cell imaging

Organelle Ca^2+^ levels were measured using the cell permeable Ca^2+^ probe Cal Red R525/650 a.m. (Cat. #20591; AAT Bioquest). The Ca^2+^ release was calculated ratiometrically between the two emission wavelengths. Cells were incubated in 5 μM Cal Red R525/650 a.m. in DMEM with 0.005% Pluronic F127 for an hour. After this, the cells were washed for 10 min in low Ca^2+^ containing HBSS to ensure complete hydrolysis of the membrane permeant acetoxymethyl (AM) ester and full release of the loaded Cal Red R525/650 a.m. Cells were then washed once more in the same HBSS before being imaged in 100 μL of HBSS. Imaging buffer for Ca^2+^ experiments comprised of ultra-low Ca^2+^ containing Hank’s balanced salt solution (HBSS; ThermoFisher Scientific) with 1 mM 4-(2-hydroxylethyl)-1-pip-eraineetha-nesulfonic acid (HEPES; Sigma), 1 mM MgCl_2_ and approximately 0.025 mM CaCl_2_ in MilliQ water. Thapsigargin (Cat. #T7458; ThermoFisher Scientific) at a concentration of 1 μM was used to measure ER Ca^2+^ content.

The Ca^2+^ images were acquired using a X-Light V3 spinning disk unit (CrestOptics) with a Lumencor Celesta Laser Engine on a Nikon Eclipse Ti2 microscope. Images were acquired using a Hamamatsu ORCA Fusion sCMOS camera. The system was operated with NIS Elements 5.21 software (Nikon). A 20x/0.75 objective was used to collect images at 10 s intervals for 40 s to establish a baseline after which Thapsigargin was added, and images were taken at 1 s intervals for 2 min to capture the peak. After this, images were captured at 5 s intervals for 3 min. The 477 nm laser line was used for exciting the Cal Red R525/650 a.m. Two emission filters 520/40nm and 700/75 nm were used for the ratio imaging.

### 2.17 Proximity ligation assay

PLA was performed on iPSCs-derived neurons using Duolink *In Situ* Starter Kit Mouse/Rabbit (Sigma-Aldrich, St. Louis, Missouri, U.S.) according to the manufacturer’s instruction. Briefly, cells were seeded in an 8-well glass chamber slide (Millicell EZ slide, Millipore) at a density of 5,000–7,000 cells. Neurons were fixed with 4% PFA for 20 min and then washed twice in 1x PBS. Permeabilization was done with 0.1% PBS-Triton-X for 30min followed by blocking using Duolink Blocking solution for 60min at 37°C using a humid chamber. After blocking, cells were incubated with VDAC (ab154856, Abcam) and IP3R (sc-377518, Santa Cruz Biotechnology) primary antibodies, diluted in in the Duolink antibody diluent, overnight at 4°C. Neurons were washed with buffer A and incubated for 1 h at 37°C with PLUS and MINUS PLA probes (Probe anti-mouse MINUS, DUO92004 and Probe anti-rabbit PLUS, DUO92002) diluted 1:5 in the Duolink antibody diluent. Coverslips were washed with buffer A and ligation and amplification steps were performed using Duolink *in Situ* Detection reagents Red for 30 and 100min respectively. Coverslips were then washed with buffer B and mounted using Duolink *In situ* mounting medium with DAPI. Images were obtained using an Andor Dragonfly spinning disc confocal microscope with a ×40 glycerol-immersion objective. Images were processed and quantified using IMARIS software and expressed as the number of PLA puncta per nucleus.

### 2.18 Statistics

Data were analyzed with GraphPad Prism (Version 10) and represented as Mean ± SEM. Comparison between two groups was performed with unpaired, 2-tailed Student’s t*-*test and among three groups with one way ANOVA. *p*-value ≤0.05 was considered statistically significant where * corresponds to ≤0.05, ** to ≤0.01, *** to ≤0.001 and **** to ≤0.0001. Each point on the graphical representation corresponds to biological repeats of the cell lines.

## 3 Results

### 3.1 CRISPR/Cas9 mediated *FXN* gene editing of GAA expansion in FRDA iPSCs rescue neuronal morphology and viability

**FIGURE 1:**
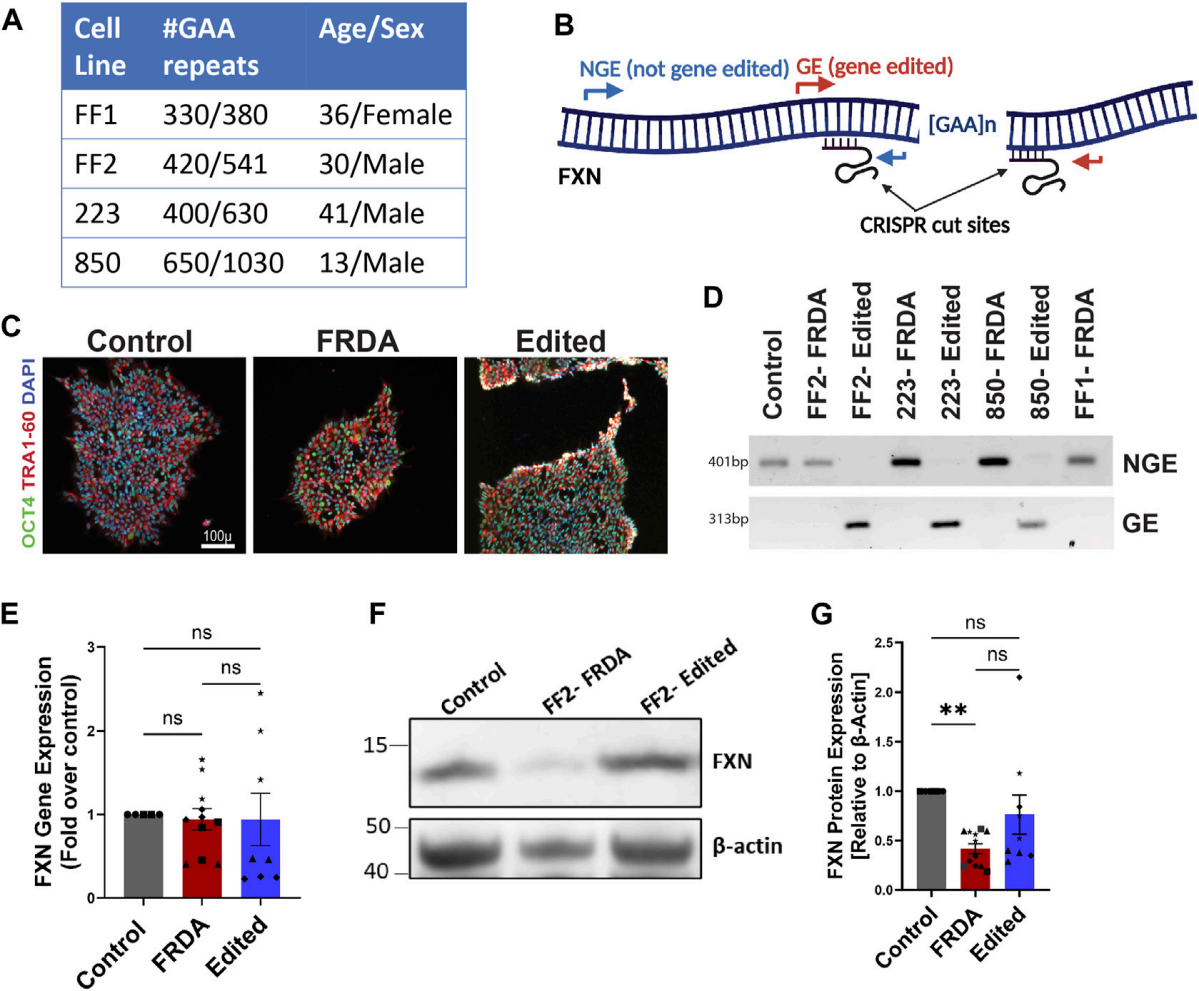
Generation of isogenic FRDA iPSC cohort using gene editing. **(A)** Four FRDA iPSC cell lines were generated with varying pathogenic GAA repeat numbers. **(B)** Schematic of dual CRISPR guide gene editing approach for GAA repeat deletion and PCR primer locations for validation **(C)** Pluripotency marker expression of resulting cell lines **(D)** Gel electrophoresis of PCR products from the GE and NGE primers identified in **(B)**. **(E)** Quantification of *FXN* mRNA expression analyzed with ddPCR. Human TBP (TATA-box binding protein) was used as reference gene. **(F)** Western blot for mature, mitochondrial FXN protein expression with β-actin as loading control. **(G)** Quantification of FXN protein expression normalized to β-actin. 2-3 biological replicates of all cell lines were used for both mRNA and protein expression studies where ●corresponds to 264 control cell line, ■ represents FF1 FRDA and E35 gene corrected cell line in the respective columns, ▲ FF2 FRDA and FF2 Edited, in respective columns, ♦ 223 FRDA and 223 Edited, in respective columns and ★ corresponds to 850 FRDA and 850 Edited lines in the respective columns. Data are represented as Mean +SEM and groups are analyzed with one-way ANOVA where *p < 0.05, **p < 0.01, ***p < 0.001, ****p < 0.0001.

We used a previously reported FRDA patient-derived iPSC line (termed FF1) together with an isogenic cell line generated through CRISPR/Cas-9 homology directed repair mediated repeat correction (termed E35) (Lai et al., 2019). To expand upon this cohort, we purchased one iPSC cell line (termed FF2: GM23913, Coriell) and generated two further FRDA iPSC cell lines from patient-derived lymphoblasts. FRDA iPSCs 223 and 850 lines were generated by genetic reprogramming of patient lymphoblasts with varying sizes of GAA expansion mutations (Figure 1A), together with a healthy control lymphoblast culture. To test our CRISPR/Cas9-mediated gene editing approach, we excised the pathogenic GAA repeat mutations in the intron 1 of *FXN* in FRDA lymphoblasts utilizing our optimized protocol with Cas9 protein and UP4-DN4 guideRNAs in a ribonucleoprotein complex (Rocca et al., 2020). Resulting iPSCs were subcloned to generate clonal gene edited or isogenic unedited FRDA model iPSCs validated by specific NGE primers flanking the 5’ end of the *FXN* intron 1 and GE primers flanking the repeats deletion as previously reported (Figure 1B) (Rocca et al., 2020). Resulting iPSCs were further validated through DNA fingerprinting as well as immunostaining for pluripotency markers (Figure 1C). Neuronal precursor cells (NPCs) generated from these iPSCs were differentiated into neurons for varying lengths of time (1, 2, 3 and 4 weeks) to identify whether these cells demonstrate FRDA associated dysfunction and if excision of the expansion mutation using our gene editing protocol improved FRDA phenotypes. We first analyzed *FXN* gene (Figure 1E) and protein (Figures 1F, G) expression in FRDA and gene-edited, differentiated neurons. The expression of *FXN* mRNA exhibited high variability among the various cell lines, with elevated mRNA levels even observed in FF1 compared to the isogenic control E35 and in 223 compared to the corresponding edited line using our CRISPR/Cas9 approach (Figure 1E; Supplementary Figure S1). However, FXN protein expression between control and FRDA lines was significantly decreased and consistently increased in each related cell line upon CRISPR/Cas9 gene editing (Figures 1F, G; Supplementary Figure S1), confirming the positive impact of our gene editing approach on FXN expression.

**FIGURE 2:**
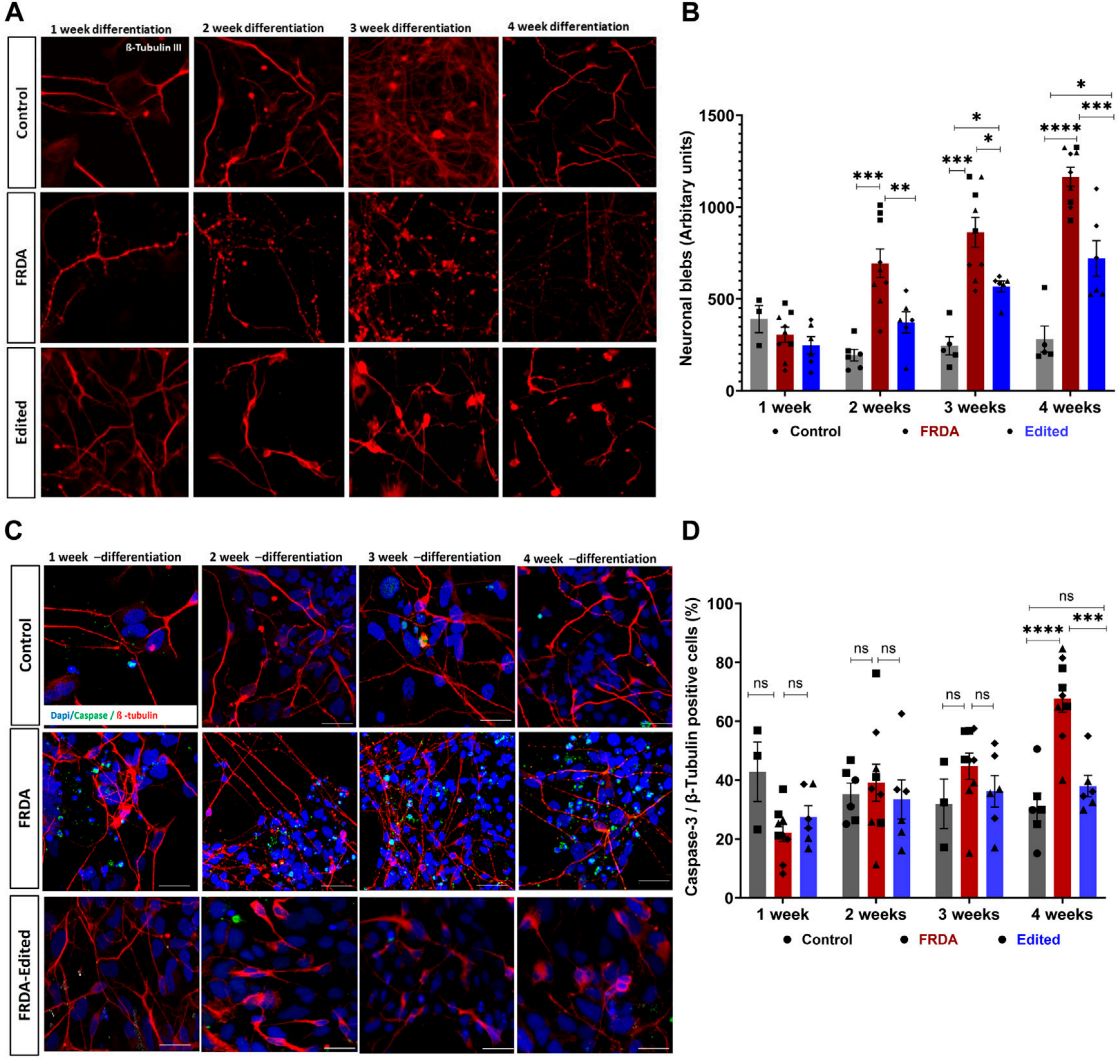
CRISPR/Cas9 gene editing prevents apoptosis of FRDA iPSC-derived neurons. **(A)** Representative confocal images of control, FRDA and gene edited neurons at different time points of differentiation (1, 2, 3 and 4 weeks) immunolabelled for neuronal marker ß-tubulin III. **(B)** Quantification of neuronal blebs across different time points; 5-8 neurons and 10 fields per cell line were used for quantification. **(C)** Representative confocal images of control, FRDA and gene edited neurons showing increasing caspase 3-positive (green) neurons from 1 week to 4 weeks. Cells were stained with ß-tubulin III (red) as neuronal marker and DAPI for nucleus. **(D)** Quantification of caspase 3, ß-tubulin positive neurons; 5-8 neurons and 10 fields per cell line were used for quantification. 2-3 biological replicates of all cell lines were used where ●corresponds to 264 control cell line, ■ represents FF1 FRDA and E35 gene corrected cell line in the graph, ▲ FF2 FRDA and FF2 Edited, in respective columns, and ♦ 223 FRDA and 223 Edited, in respective columns. Data are represented as Mean ± SEM and groups are analyzed with one-way ANOVA where *p > 0.05, **p < 0.01, ***p < 0.001, ****p < 0.0001. Scale bar, 50 μm.

The morphology of FRDA neurons, analyzed using β-tubulin III immunostaining, did not display morphological defects at 1 week of differentiation, but exhibited progressive abnormalities at subsequent time points. We observed impaired neurite connectivity and significantly increased numbers of plasma membrane blebbing at 2, 3 and 4 weeks (Figures 2A, B). Interestingly, the 850 FRDA neurons, which carried the longest GAA repeats (Figure 1A), displayed aggravated blebbing as early as 2 weeks compared to neurons from other cell lines with shorter expansions, with no viable neurons at 3 and 4 weeks for subsequent studies (Supplementary Figure S2). While the gene edited 850 neurons demonstrated trends of improved *FXN* gene and protein expression compared to respective FRDA neurons (Supplementary Figure S1), owing to the dramatic structural abnormalities in the FRDA 850 neurons (Supplementary Figure S2), these lines were excluded from microscopic studies at all time points. Dendritic blebbing is a feature of apoptotic neurons (Park et al., 1996; Jaworski et al., 2011; Erturk et al., 2014) and neurodegeneration (Kweon et al., 2017) and is mediated by caspase-3 activation that promotes cytoskeletal reorganization (Coleman et al., 2001; Erturk et al., 2014). Immunostaining for Caspase 3 revealed an increased number of active caspase 3-positive neurons overtime in the FRDA neurons (Figures 2C, D). Isogenic neurons generated from our gene editing approach displayed morphology similar to unrelated control neurons, with significantly reduced neuronal blebbing and caspase 3-positive cells compared to diseased neurons. These results demonstrate that FRDA iPSC-derived neurons display a strong disease phenotype and that our gene editing approach is effective in decreasing apoptotic response and preserving neuronal morphology.

### 3.2 FRDA neurons display impaired mitochondrial structure and increased oxidative stress, which are rescued by *FXN* gene editing

**FIGURE 3:**
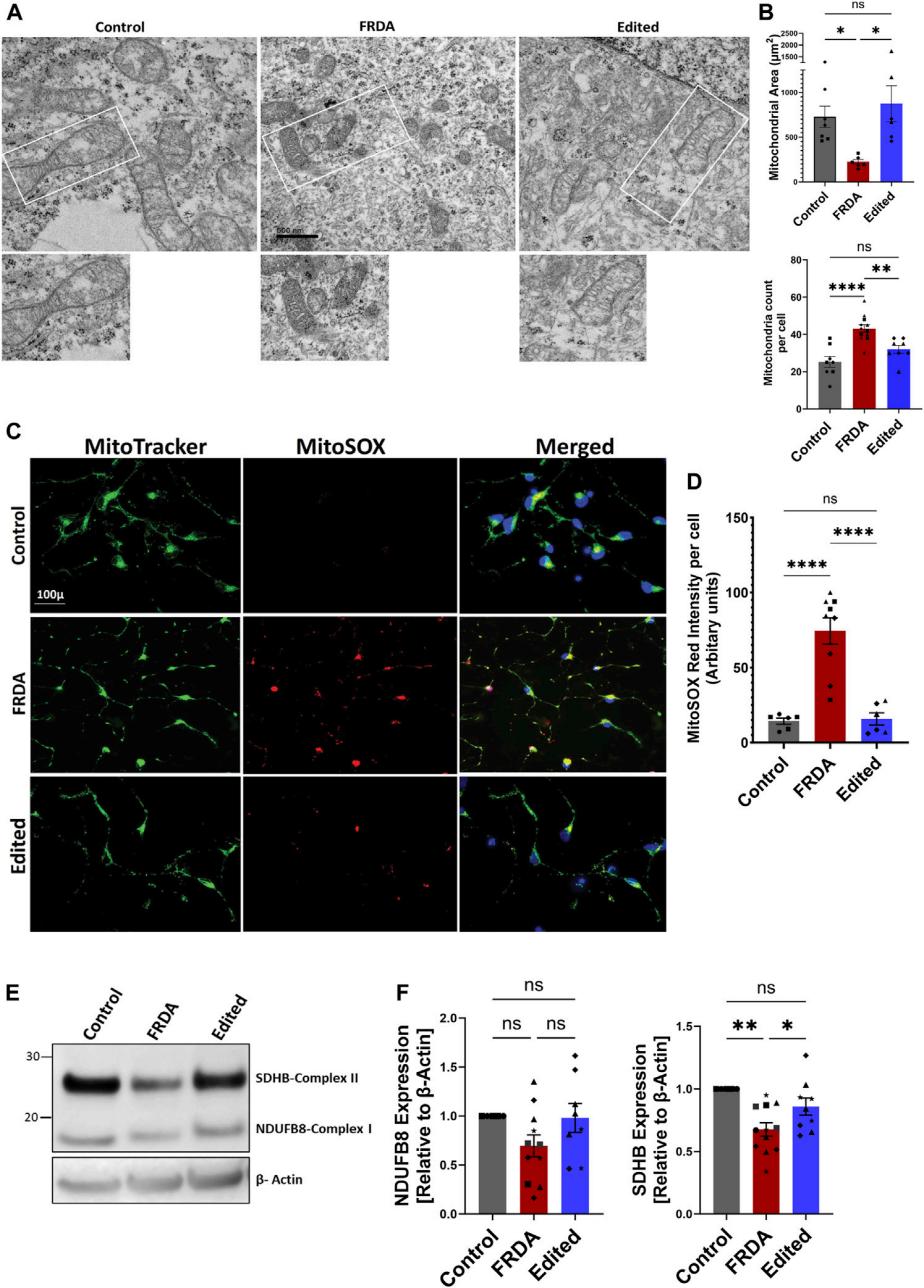
CRISPR/Cas9 mediated FXN gene editing improves mitochondrial structure and function in 2-week differentiated iPSC-derived FRDA neurons. **(A)** Representative images of transmission electron microscopy of mitochondria in control, FRDA and gene edited neurons at 2-week of differentiation (Scale bar 500 nm). **(B)** Quantification of mitochondrial numbers per cell and mitochondria area in control, FRDA and gene edited neurons. n = 3-4 cells for each cell line were used for quantification. **(C)** Representative quantification of mitochondrial superoxide levels analyzed with MitoSOX Red. Nuclei were stained with Hoechst 33342. **(D)** Quantification of MitoSOX Red fluorescence intensity per cell; 5-8 neurons per field were analyzed for each cell line and data was normalized to fluorescence intensity per cell. **(E)** Representative immunoblotting for expression of the mitochondrial electron transport chain proteins NDUFB8 and SDHB. **(F)** Quantification of expression of NDUFSB8 and SDHB as seen by Western blot analysis **(E)** and normalized to β-actin as a loading control. 2-3 biological replicates of all cell lines were used where ●corresponds to 264 control cell line, ■ represents FF1 FRDA cell line in the graph, ▲ FF2 FRDA and FF2 Edited, in respective columns, ♦ 223 FRDA and 223 Edited, in respective columns and ★ corresponds to 850 FRDA and 850 Edited lines in the respective columns. Data are represented as Mean +SEM and groups are analyzed with one-way ANOVA where *p < 0.05, **p < 0.01, ***p < 0.001, ****p < 0.0001.

Because mitochondrial oxidative stress and electron transport chain functions are impaired in FRDA (Bradley et al., 2000; Heidari et al., 2009), we next analyzed mitochondrial structure and oxidative stress response in iPSC derived FRDA neurons and potential rescue by gene editing. All experiments, henceforth, were conducted on 2-week differentiated neurons as this time point demonstrated a balance between sufficient neuronal morphological changes to identify the FRDA phenotype while retaining acceptable viability (Figure 2). Electron microscopic analysis of FRDA neurons displayed a high number of circular, fragmented mitochondria with degenerated cristae structure (Figures 3A, B). The mitochondrial electron transport chain complexes span the mitochondrial outer and inner membrane (Kuhlbrandt, 2015). Damaged mitochondrial cristae directly impact the function of these electron transport chain super complexes. Further, mitochondrial fission (Itoh et al., 2013) and cristae remodeling (Garrido et al., 2006) are hall marks of cell apoptosis, strengthening our observation that dysfunctional mitochondria in FRDA neurons promotes cell death, potentially by releasing pro-apoptotic factors, and thus failing to sustain neuronal viability beyond 2 weeks. In contrast, CRISPR/Cas9 gene-edited neurons displayed intact mitochondrial outer and inner membrane, tubular and networked structures with well-organized cristae, similar to unrelated control neurons (Figures 3A, B).

Because frataxin deficiency induces a state of oxidative stress by crippling the mitochondrial respiratory complex, and gene-editing improved mitochondrial morphology, we next analyzed reactive oxygen species (ROS) levels in FRDA, and gene edited neurons using MitoSOX Red, a dye that preferentially stains mitochondrial superoxides. At 2 weeks of differentiation, we observed a significant increase in mitochondrial superoxide levels in FRDA neurons compared to both the gene edited and healthy neurons (Figures Figure3C–D). ROS levels are highly dependent on electron leak from mitochondrial respiratory complexes, and both complex I and II activity and protein levels are affected in FRDA (Bradley et al., 2000; Heidari et al., 2009; Huichalaf et al., 2022; Sanz-Alcázar et al., 2023). We thus analyzed the protein expression pattern of NADH:ubiquinone oxidoreductase subunit B8 (NDUFB8) of Complex I, and succinate dehydrogenase complex iron sulfur subunit B (SDHB) of complex II (Figures 3E, F). FRDA neurons demonstrated a trend of downregulation of NDUFB8 protein expression that was improved in gene editing neurons, and a significant decrease in SDHB compared to healthy and gene edited neurons. Taken together, these data demonstrate that FRDA neurons carry dysfunctional mitochondria that exhibit increased oxidative stress, and gene editing rescues both mitochondrial structural and response to oxidative stress of the FRDA neurons.

### 3.3 Gene editing restores endoplasmic stress response genes in FRDA neurons

**FIGURE 4:**
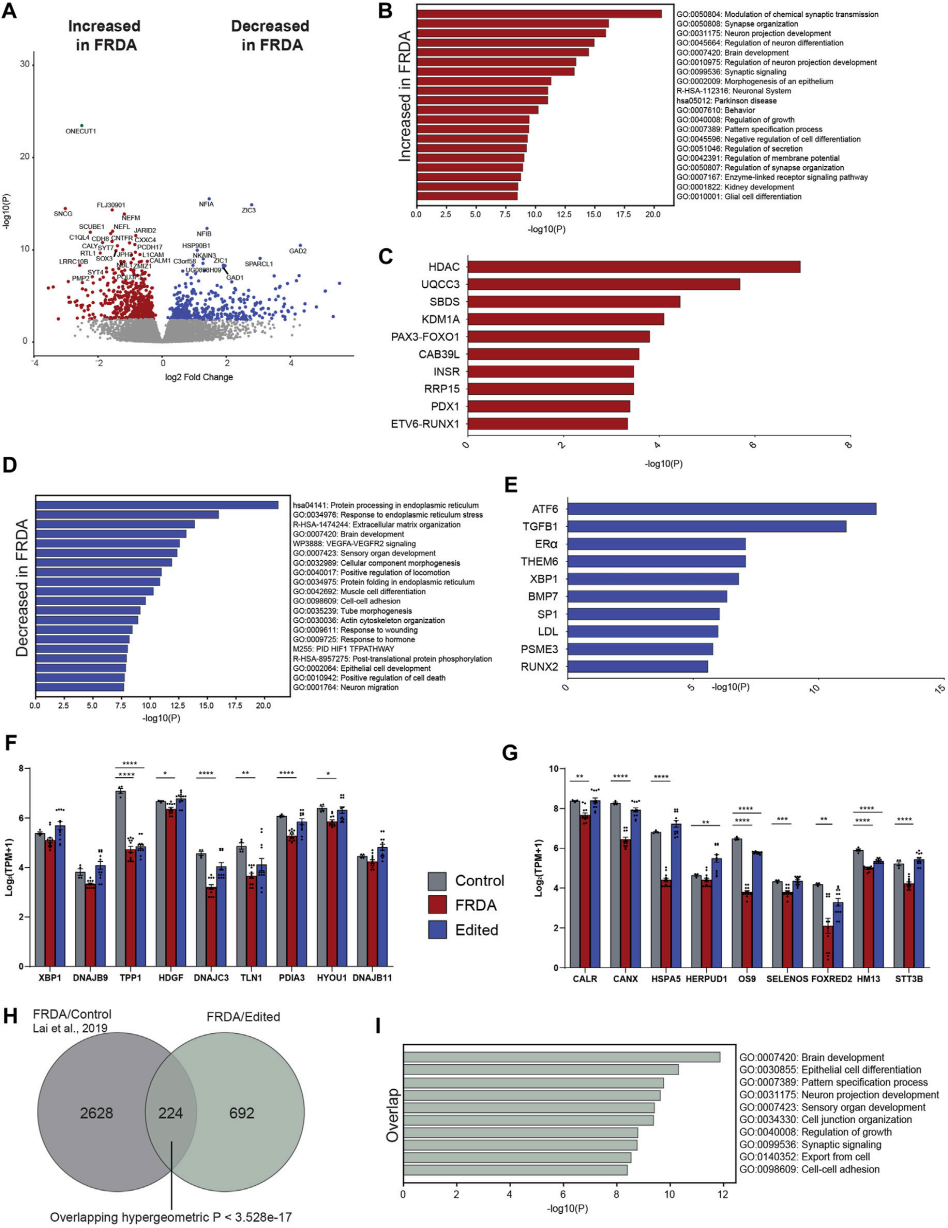
Gene editing to remove the detrimental FRDA GAA intronic repeat alters the transcriptome profile of FRDA neurons. **(A)** Volcano plot of differential gene expression delineating genes that are increased (DEG, red) and decreased (DEG, blue) in FRDA as compared to gene edited neurons from the same individuals (916 DEGs). **(B)** Gene ontology of DEGs decreased in FRDA neurons compared to gene edited neurons. **(C)** Ingenuity pathway analysis (IPA) of upstream regulators decreased in FRDA neurons **(D)** Gene ontology of genes increased in FRDA neurons compared to gene edited controls. **(E)** IPA analysis of genes increased in FRDA neurons. **(F)** Bar charts of differentially expressed genes related to XBP1 signaling and **(G)** ER associated protein degradation (ERAD) showing correction with CRISPR repeat deletion (Edited) when compared to isogenic control with homology directed GAA repeat repair (one way ANOVA, *p < 0.05, **p < 0.01, ***p < 0.001, ****p < 0.0001). **(H)** Venn diagram comparing DEGs from FRDA neurons and isogenic repeat corrected controls (Lai et al., 2019) with the DEGs here showing statistically significant overlap. **(I)** Gene ontology of overlap genes from **(G)**.

**FIGURE 5:**
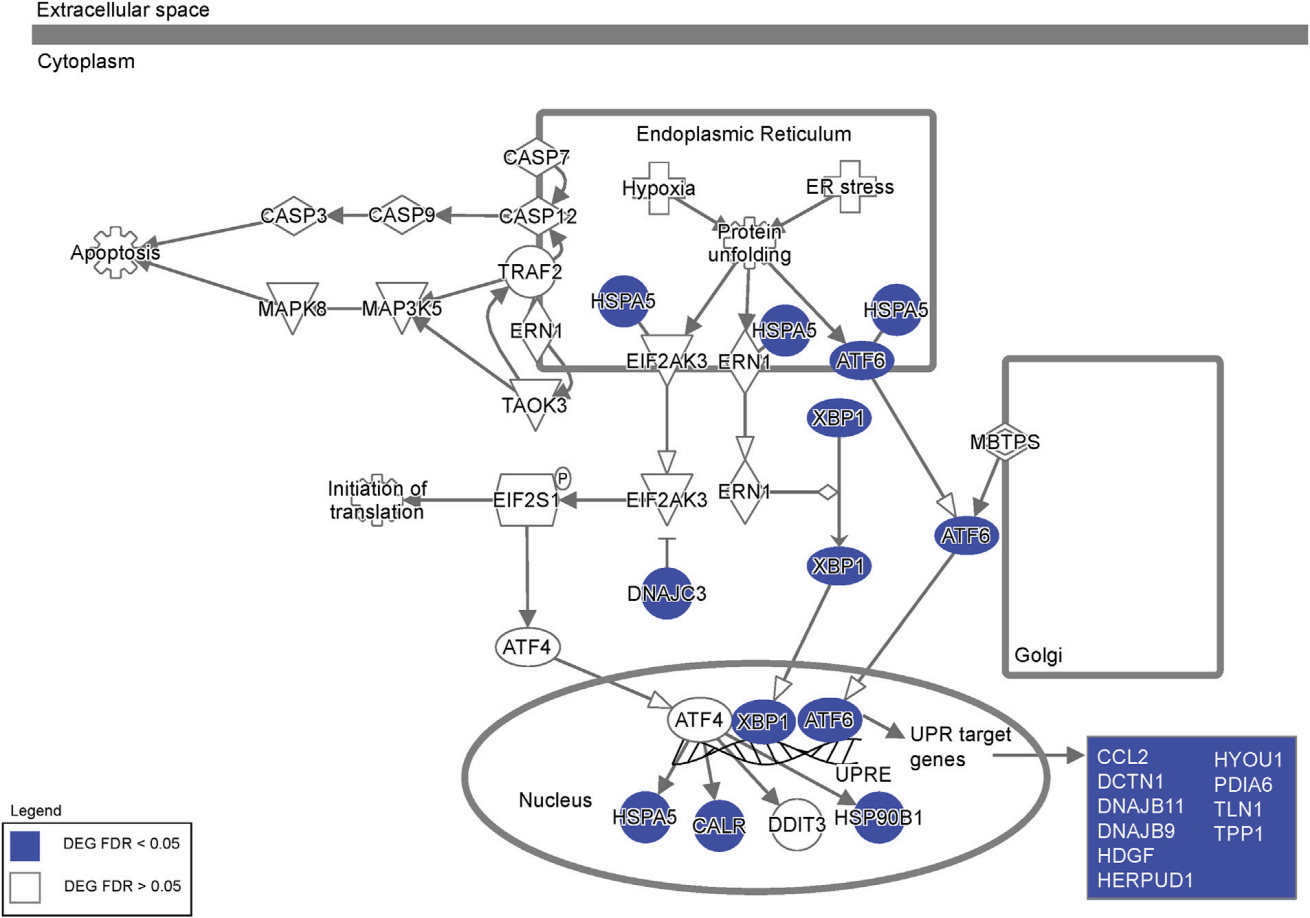
Dysregulation of endoplasmic reticulum stress pathway genes. Ingenuity Pathway Analysis of the ER stress pathway highlighting differentially expressed genes in FRDA neurons (labeled in blue) representing extensive dysregulation of ER stress in FRDA neurons.

To investigate the effects of *FXN* gene editing on global gene expression and the pathways involved in loss of cellular homeostasis in FRDA, we performed transcriptomic profiling on 2 weeks differentiated FRDA and gene edited neurons. E35 isogenic neurons were used as a control comparison for the analysis. *FXN* transcripts were below 4 transcripts per million (TPM), the lower threshold for statistical analysis. A comparison between FRDA and gene edited neurons identified a total of 381 upregulated and 534 downregulated gene transcripts between FRDA and edited neurons (Figure 4A and Supplementary Figure S3A, B). Gene ontology analysis identified increased expression of genes involved in synaptic transmission and organization (Figure 4B) in FRDA and Ingenuity Pathway Analysis (IPA) displayed dysregulation of ubiquinol cytochrome C (UQCC3) associated mitochondrial disease pathways (Figure 4C). Pathways related to epigenetic signaling, and synapse maturation, such as *HDAC* (Akhtar et al., 2009; Sada et al., 2020) and *KDM1A* (Ambrosio and Majello, 2018) were upregulated in FRDA; inhibitors of these genes have been explored for neurodegenerative diseases, including FRDA (Rai et al., 2010; Soragni et al., 2014; Polak et al., 2016; Soragni and Gottesfeld, 2016). Conversely, genes downregulated in FRDA neurons compared to isogenic gene edited neurons included those involved in endoplasmic reticulum (ER), protein processing and the response to ER stress (Figure 4D). Both ATF6 and XBP1 were identified as upstream regulators lost in FRDA (Figure 4E). The connection between mitochondrial dysfunction and ER stress has not been deeply investigated in FRDA. This analysis identified loss of expression of multiple genes associated with the XBP1 pathway (Figure 4F) and ER associated protein degradation (ERAD) (Hetz, 2012) in FRDA that was overall improved or normalized in gene edited neurons (Figure 4G). FRDA neurons displayed significantly downregulated expression of genes such as calreticulin (*CALR*), calnexin (*CANX*), Binding immunoglobin protein (*HSPA5*) and Protein Disulfide Isomerase Family A member 3 (*PDIA1*) that work, individually or in tandem, to suppress protein transcription and translation to mitigate ensuing stress. Lastly, we compared our transcriptomic data to the only published FRDA iPSC neuronal datasets (Lai et al., 2019), which derived neurons utilizing a different differentiation protocol. We found statistically significant overlap between differentially expressed genes (Figure 4H), overlapped genes primarily involving neuronal maturation (Figure 4I). Altogether, the transcriptomic analysis identified the interaction between ER stress, the unfolded protein response, and calcium homeostasis as key pathways dysregulated in FRDA (Figure 5). These pathways appear comparable to the healthy controls in the gene editing neurons.

### 3.4 Gene editing improves endoplasmic reticulum morphology and ER-mitochondria contacts in FRDA neurons

**FIGURE 6:**
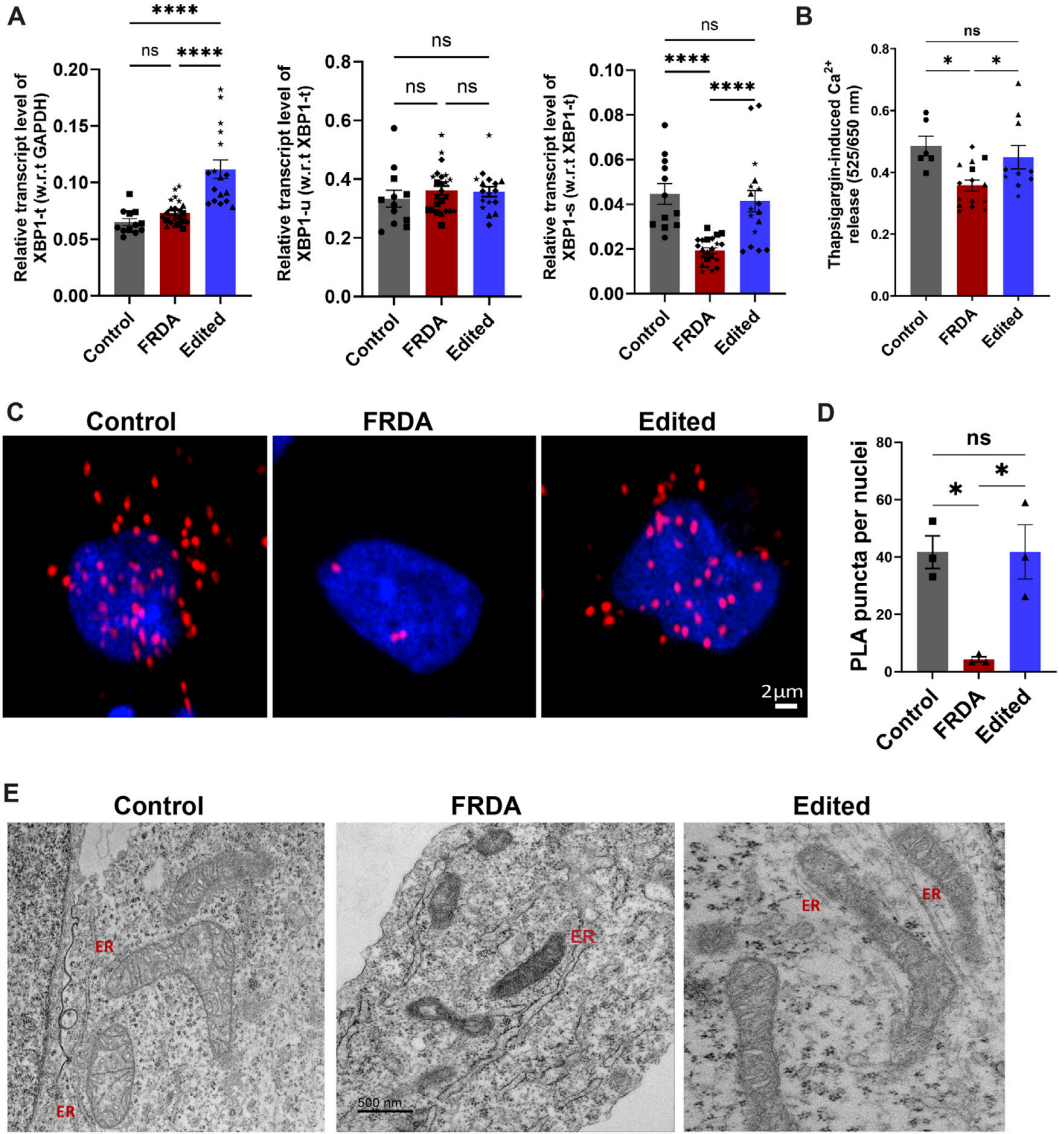
Restoration of mitochondrial-ER contacts in CRISPR-Cas9 gene edited iPSC-neurons. **(A)** qRT-PCR based validation of XBP1 expression where Total XBP1(XBP1-t) was normalized to GAPDH as internal control. Unspliced XBP1 (XBP1-u) and active, spliced XBP1 (XBP1-s) are normalized to (XBP1-t). **(B)** Thapsigargin (1 µM) mediated ER-calcium release analyzed with Cal Red R525/650 a.m. Quantification was performed on 10–20 neurons in 3-4 different batches of neuronal differentiation per cell line. All cell lines were used for both studies and in the graph, ●corresponds to 264 control cell line, ■ represents FF1 FRDA and E35 cell line, in respective columns, ▲ FF2 FRDA and FF2 Edited, in respective columns, ♦ 223 FRDA and 223 Edited, in respective columns and ★ corresponds to 850 FRDA and 850 Edited lines in respective columns. **(C)** Representative confocal images from proximity ligation assay (PLA) depicting VDAC and IP3R interactions in control, FRDA and gene-edited neurons. **(D)** Quantitative analysis of PLA puncta per nuclei shows a strong decrease of mitochondria-ER contacts in FRDA neurons compared to control line. Quantification was performed on 60–80 neurons per cell line from three independent experiments where E35 was used as control (■), FF2 as representative of FRDA and FF2 Edited as representative of CRISPR/Cas9 edited cell line (▲). PLA puncta are shown as the number of puncta/nuclei. **(E)** Representative transmission electron microscopic images from control, FRDA and gene edited neurons showing no mitochondrial-ER contact sites and lack of ER structures in FRDA neurons. ER-Endoplasmic reticulum; Scale bar 500 nm. Data are represented as Mean +SEM and groups are analyzed with one-way ANOVA where *p < 0.05, **p < 0.01, ***p < 0.001, ****p < 0.0001.

The endoplasmic reticulum is the calcium store of the cell and undergoes dynamic morphological changes necessary for protein folding. To functionally validate dysregulation in ER homeostasis in FRDA neurons with subsequent correction through gene editing, we analyzed a representative ER-stress responsive gene, X-box-binding protein 1 (*XBP-1*) and calcium release in the presence of ER stressor, thapsigargin. *XBP-1* is a major regulator of ER stress response and was identified as an upstream regulator in our transcriptomic analysis (Figure 4E) and undergoes alternative splicing to generate two isoforms, unspliced (*XBP-1u*) and spliced XBP-1 (*XBP-1s*). While *XBP-1u* is inactive and undergoes rapid proteasomal degradation (Calfon et al., 2002; Tirosh et al., 2006), transcriptional activity of *XBP-1s* in the nucleus activates genes for combating protein accumulation and alleviating ER stress (Yoshida et al., 2001; Margariti et al., 2013). FRDA neurons displayed significantly reduced expression of *XBP-1s* compared to gene edited and control neurons while there was no significant difference in *XBP-1u* (Figure 6A). In line with these results, when FRDA neurons were treated with thapsigargin, an ER stressor that promotes calcium leak, these cells had significantly attenuated ER calcium release compared to control neurons. In contrast, gene edited neurons had improved calcium release compared to FRDA neurons (Figure 6B). To understand these responses in FRDA and gene edited neurons in the context of mitochondrial structure and function, we next analyzed changes in mitochondria-ER contact sites that are critical for maintaining calcium homeostasis. Mitochondria-associated ER membranes (MAMs) are one of the best-characterized inter-organelle interactions where the two organelles are physically tethered to each other via Inositol-1,4,5-triphosphate receptor (IP3R) on the ER and Voltage-dependent anionic channel (VDAC) on the mitochondria. *In situ* proximity ligation assay (PLA) demonstrated marked and significant reduction in the number of PLA positive puncta per cell in FRDA neurons compared to control neurons (Figures 6C, D). Importantly, gene edited neurons displayed significantly increased and preserved interaction sites compared to FRDA and control neurons, respectively. Ultrastructural analysis of these contact sites in FRDA neurons displayed a dramatic lack of ER structures that was in contrast preserved in gene edited neurons (Figure 3D). Altogether, based on the enriched transcriptomic profile ER stress response, preservation of ER morphology and calcium homeostasis, *FXN* gene editing improves overall cellular homeostasis in FRDA neurons, contributing to improved cell survival.

## 4 Discussion

In the present study, we investigated and validated the efficacy of our gene editing approach in improving mitochondrial and cellular homeostasis utilizing a novel cohort of FRDA iPSC derived neurons. We identified novel pathological signatures of defective ER morphology and ER-mitochondrial contacts that are rescued in gene edited neurons. These contacts in gene edited neurons contribute to augmented ER stress-response and synergistic communication between mitochondria and the ER.

The etiology of FRDA pathophysiology is primarily attributed to chronic mitochondrial dysfunction and ensuing oxidative stress that promotes neurodegeneration (Lupoli et al., 2018). While the function of FXN is still unclear, it is widely reported to participating in Fe-S biosynthesis (Fox et al., 2019) and thus deficiency in FXN augments redox imbalance at the electron transport chain. Mitochondria play a critical role in apoptosis regulation and mitochondrial structural integrity, partially dependent on redox homeostasis, ensure internalization of pro-apoptotic factors (Sivakumar et al., 2017; Kondadi et al., 2020). In the presence of chronic stress, such as accumulating oxidative stress in FRDA, mitochondria undergo increased fragmentation and release apoptotic factors into the cytosol that activates caspase signaling, leading to cell death (Youle and Karbowski, 2005; Sivakumar et al., 2020). Maturation of FRDA iPSC- derived neurons up to 4 weeks displayed progressive apoptosis that is dependent on GAA expansion length and accumulating mitochondrial dysfunction. The 850 FRDA neurons, carrying the longest repeat length in our cohort (650/1030 repeats), had compromised viability by 2 weeks of differentiation compared to other FRDA lines with shorter repeat lengths. FRDA neurons also demonstrated increased mitochondrial superoxide levels along with fragmented mitochondrial ultrastructure with poorly preserved cristae invaginations, corroborating worsening mitochondrial dysfunction in FRDA. Thus, our iPSC-derived neurons recapitulate *in vitro* FRDA disease phenotypes reported previously (Hick et al., 2013; Mincheva-Tasheva et al., 2014; Igoillo-Esteve et al., 2015; Codazzi et al., 2016), and are suitable models for validating the efficiency of our gene-editing approach.

We generated isogenic iPSCs utilizing our targeted gene editing approach to remove the GAA expansion in intron 1 of *FXN* (Rocca et al., 2020), and validated its efficacy by differentiating these iPSCs to neurons. Differentiated neurons displayed morphologically well-preserved neurites up to 4 weeks of maturation with tubular mitochondrial structures, well-organized cristae invaginations, altogether indicative of healthy mitochondrial morphology (Mandal and Drerup, 2019), and resulting in reduced cell apoptosis. Despite the early-onset and dramatic structural abnormalities in the 850 FRDA lines carrying the longest GAA expansion in our cohort, the respective gene edited line demonstrated trends of increased *FXN* gene and protein expression, confirming that our CRISPR/Cas9-mediated gene editing has positive impacts on neuronal health, irrespective of the expansion length. To understand the mechanism of neuronal rescue by gene-edited, we utilized 2 week differentiated neurons that ensured a balance between neuronal differentiation and early apoptosis for transcriptomics analysis. Overlapping transcriptomic profiles with previous reports (Lai et al., 2019) demonstrated the reproducibility and validity of both our FRDA model and the gene editing correction. As such, pathways associated with epigenetic changes were predicted upstream regulators disrupted in FRDA neurons and normalized in the gene edited cells. Epigenetics as a therapeutic approach for FRDA has been extensively explored and notably, HDACi (2-aminobenzamide–type histone deacetylase inhibitors) increased *FXN* expression in peripheral blood mononuclear cells of patients enrolled in a Phase I clinical trial with a promising safety profile (Rai et al., 2010; Soragni et al., 2014; Polak et al., 2016; Soragni and Gottesfeld, 2016). While we have not performed chromatic immunoprecipitation studies to validate the histone modification status around the *FXN* landscape, it is possible that gene-editing mediated excision of the expansion reduced the repressive state of heterochromatin or formation of DNA-DNA/DNA-RNA triplexes, structures that are associated with transcriptional repression of *FXN* (Gottesfeld et al., 2013). Mazzara et al. reported that complete excision of GAA expansion of nearly 10 kb on intron 1, with regulatory elements, was required to overcome repressive histone hallmarks, in FRDA DRG organoids (Mazzara et al., 2020). However, we have demonstrated that our CRISPR/Cas9 guide RNA pair, UP4-DN4, spanning the GAA expansion mutation, mt-binding site, and E-box regulatory sequences of *∼*2 kb was effective and sufficient for functional rescue (Rocca et al., 2020). This excision and loss of regulatory sequences did not affect *FXN* transcription in FRDA lymphoblasts and gene edited CD34^+^ HSPCs retained normal hematopoietic reconstitution potential, *in vitro* and *in vivo* (Rocca et al., 2020). The gene-edited iPSC-derived neurons in this study, carrying varying GAA repeat lengths, corroborated these findings of improved cellular and mitochondrial response, and demonstrate the efficacy of the editing process, even in the presence of challenging access to the chromatin.

Interestingly, genes associated with endoplasmic reticulum stress response, protein folding, and processing had predominant suppressed signatures in the transcriptomic profile of iPSC-derived neurons. Mitochondria and endoplasmic reticulum physically tether to form MAMs involved in calcium homeostasis and lipid synthesis and transfer (Lee and Min, 2018; Yang et al., 2020). Imbalance in these sites ultimately hampers protein quality control. Dysregulation in these contacts have been previously reported in FRDA in a neuroblastoma cell line and in a *drosophila* model (Rodriguez et al., 2020) where the FRDA phenotype was mimicked with *FXN* hairpins. We demonstrated similar findings of reduced contacts sites in patient iPSC derived FRDA neurons using proximity ligation assay and electron microscopy that were rectified upon gene editing of the pathogenic repeat. To further understand whether loss of these contact sites was due to canonical mitochondrial structural abnormalities associated with FRDA, we looked at ultrastructural morphology of both the ER and mitochondria in FRDA neurons and observed strikingly compromised ER structures, in addition to mitochondrial abnormalities. ER morphology and its distribution are a dynamic phenomenon within the cell and are essential for protein synthesis, folding and post-translation modifications (Perkins and Allan, 2021). Accumulation of misfolded proteins or their aggregates triggers ER stress-response called “unfolded protein response” (UPR) activating genes such as *IRE1*, *ATF6*, *ATF4, XBP-1* that support suppression of transcription and translation to process degradation of accumulated proteins (Hetz, 2012). Chronic ER stress translates UPR to apoptotic signals and has been reported in several neurodegenerative disorders (Bhattarai et al., 2021). We validated the expression of splice forms of the transcription factor, *XBP-1* (X-box-binding protein 1)*,* as a representative gene of suppressed ER stress. Downregulation of XBP-1 is associated with neurodegeneration (Valdes et al., 2014; McLaughlin et al., 2018) and FRDA neurons display downregulation of the active spliced variant of *XBP-1* that was reverted in gene edited neurons. Functional implications of both downregulated *XBP-1s* and limited ER-mitochondria contact sites were reflected in attenuated calcium release from the ER in FRDA neurons and was improved in gene edited neurons. Considering both the structural damage to the ER and the transcriptomic profile indicating the suppression of ER stress response genes such as *XBP-1*, it is reasonable to propose that protein toxicity may induce apoptosis in FRDA neurons. In contrast, the gene-editing leading to the excision of GAA expansion mutation in *FXN* supports sustaining ER morphology, dynamics, and protein processing, and these data showed that our gene editing approach restored ER-mitochondrial contacts and improved mitochondrial homeostasis, which plays a crucial role in neuronal cell survival.

Altogether, the results of this study represent crucial efficacy profile of our CRISPR/Cas9 approach in a disease relevant model of FRDA. Neurons generated from FRDA iPSCs effectively recapitulate mitochondrial dysfunction of the disease and validate the potential of *FXN* gene editing in improving mitochondrial and cellular homeostasis. Furthermore, gene editing improved cellular response to protein toxicity mediated by ER structural damage, either in tandem or downstream of mitochondrial rescue, enhancing our approach as an effective strategy for therapeutic intervention for Friedreich’s ataxia.

## Data Availability

The RNA sequencing datasets from FRDA, edited, and isogenic control for this study can be found in the Gene Expression Omnibus (GEO). The accession number is GSE244886.
