# Supplementary material for: Gene editing improves endoplasmic reticulum-mitochondrial contacts and unfolded protein response in Friedreich’s ataxia iPSC-derived neurons
Source: Front Pharmacol. 2024 Feb 14;15:1323491. doi: 10.3389/fphar.2024.1323491 (PMC10899513; doi:10.3389/fphar.2024.1323491)
Supplement: Supplementary file 1 [file Table1.DOCX]

**Table S1.** Human primer sequences for qPCR. All primers were reconstituted at 100 μM and used at a working concentration of 5 μM.

| Gene | Full Name | Purpose | Direction (5’-3’) | Sequence |
| --- | --- | --- | --- | --- |
| *GAPDH* | glyceraldehyde-3-phosphate dehydrogenase | qPCR Housekeeping | Forward  Reverse | TCAAGGCTGAGAACGGGAAG  CGCCCCACTTGATTTTGGAG |
| Total *XBP1* (t*XBP1*) | X-box binding protein 1 [total XBP1 (tXBP1)] | mRNA Expression | Forward  Reverse | TGAAAAACAGAGTAGCAGCTCAGA  CCCAAGCGCTGTCTTAACTC |
| Unspliced *XBP1* (u*XBP1*) | X-box binding protein 1 [Unspliced XBP1 (uXBP1)] | mRNA Expression | Forward  Reverse | CAGACTACGTGCACCTCTGC  CTGGGTCCAAGTTGTCCAGAAT |
| Spliced *XBP1* (s*XBP1*) | X-box binding protein 1 [Spliced XBP1 (sXBP1)] | mRNA Expression | Forward  Reverse | GCTGAGTCCGCAGCAGGT  CTGGGTCCAAGTTGTCCAGAAT |
