# Supplementary figures and images for: Gene editing improves endoplasmic reticulum-mitochondrial contacts and unfolded protein response in Friedreich’s ataxia iPSC-derived neurons
Source: Front Pharmacol. 2024 Feb 14;15:1323491. doi: 10.3389/fphar.2024.1323491 (PMC10899513; doi:10.3389/fphar.2024.1323491)

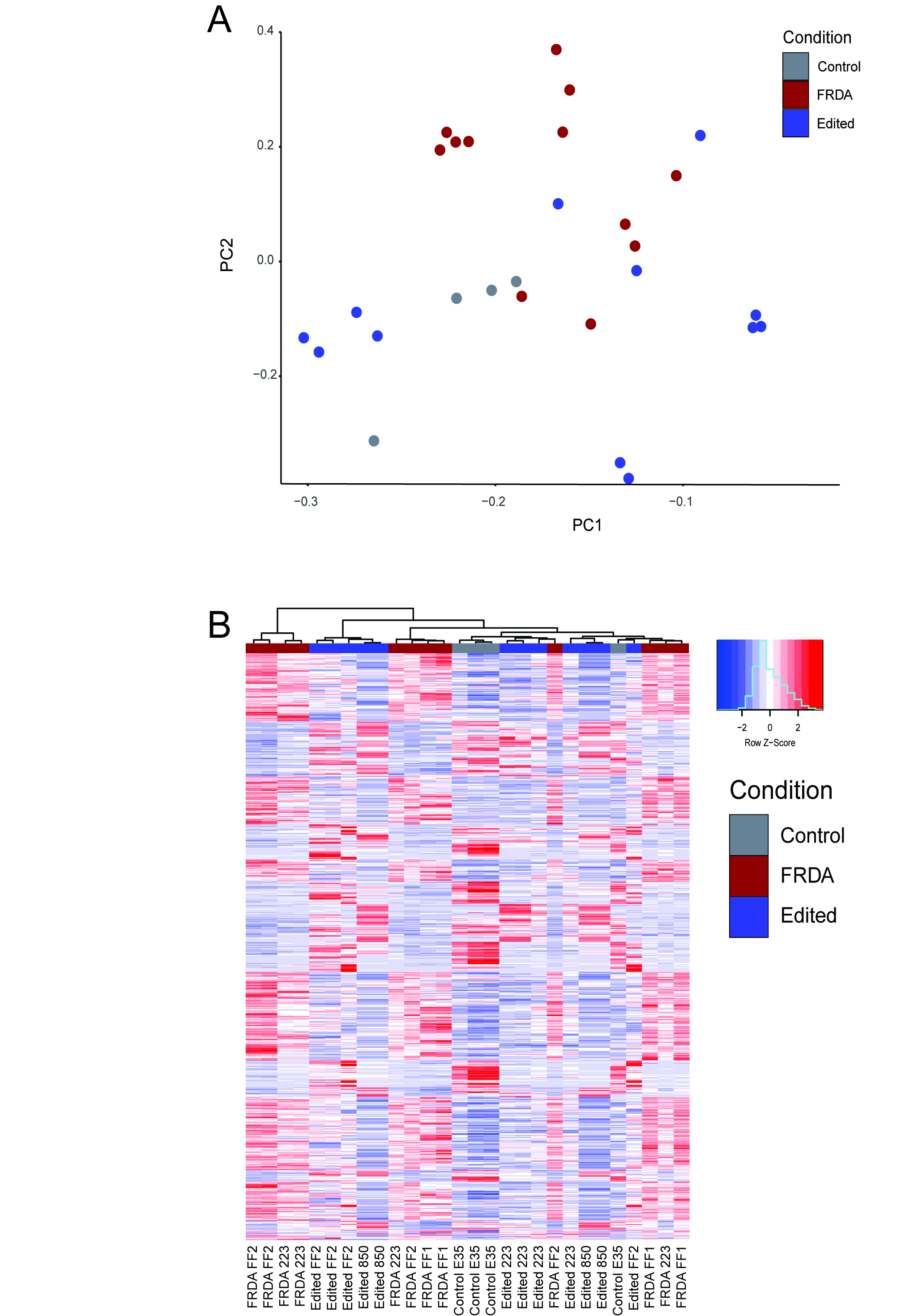

Supplement: Supplementary file 2 [file Image3.TIF]

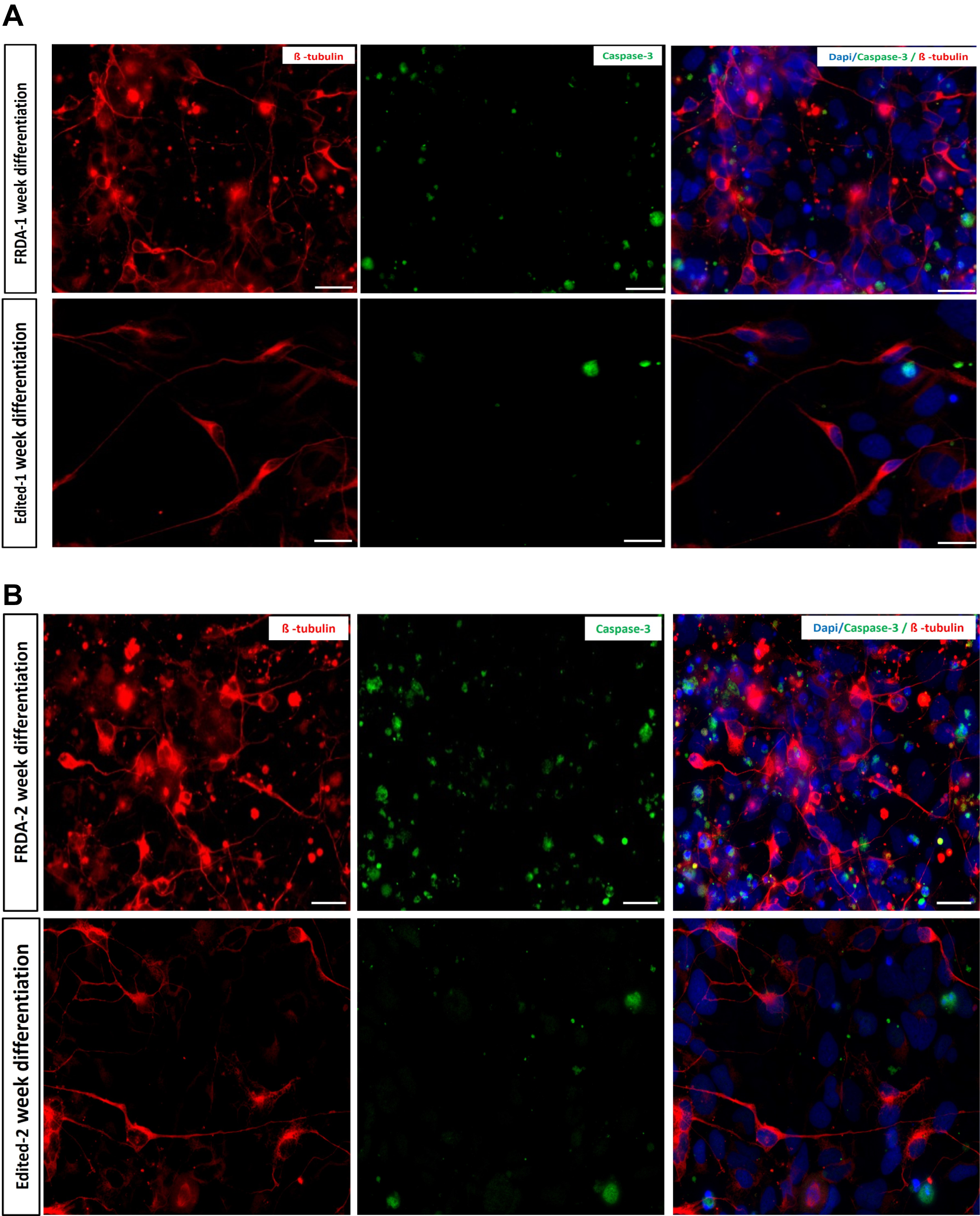

Supplement: Supplementary file 3 [file Image2.TIF]

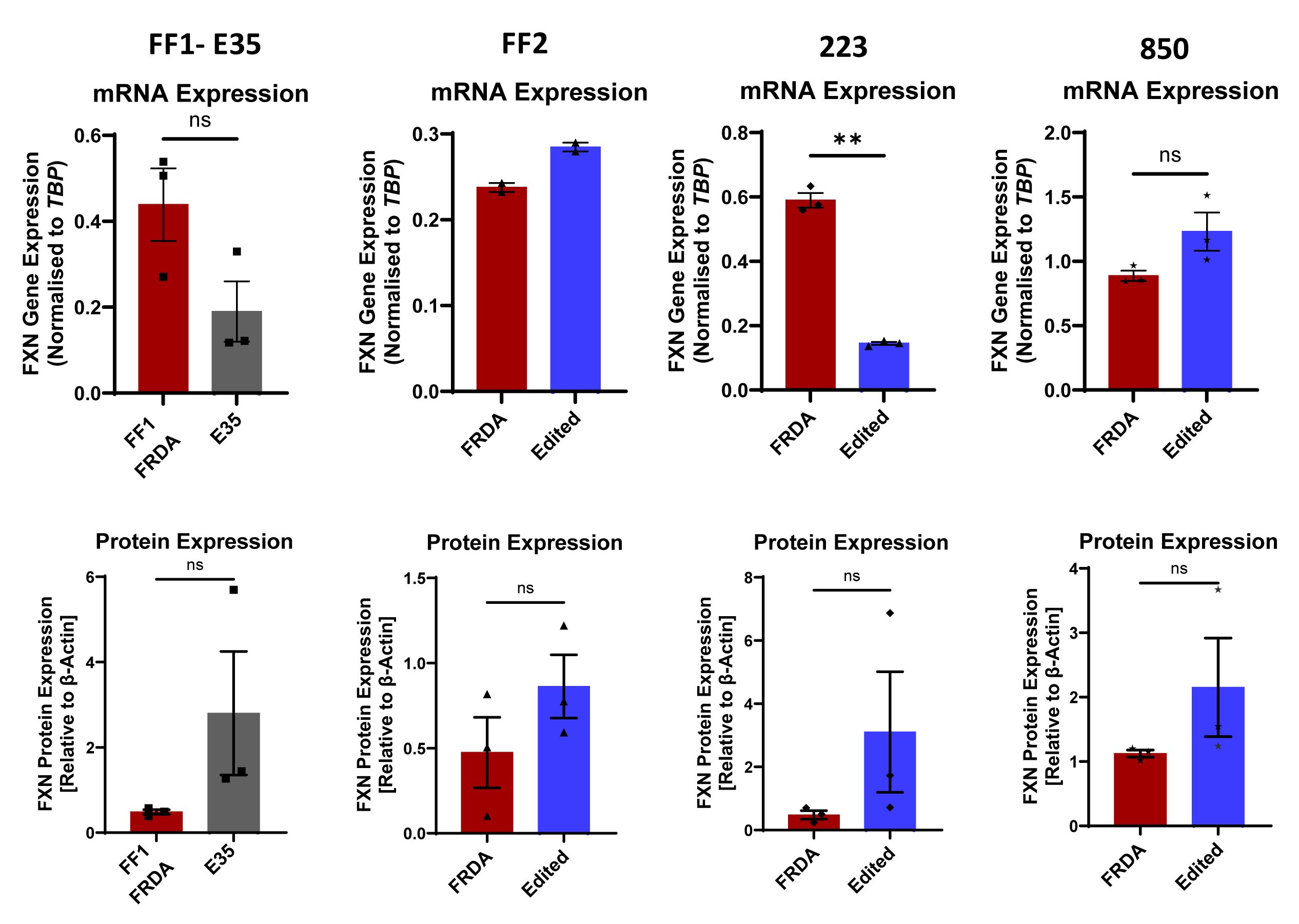

Supplement: Supplementary file 4 [file Image1.TIF]
