# Supplementary material for: Gene editing improves endoplasmic reticulum-mitochondrial contacts and unfolded protein response in Friedreich’s ataxia iPSC-derived neurons
Source: Front Pharmacol. 2024 Feb 14;15:1323491. doi: 10.3389/fphar.2024.1323491 (PMC10899513; doi:10.3389/fphar.2024.1323491)
Supplement: Supplementary file 5 [file DataSheet1.docx]

**Gene editing improves Endoplasmic reticulum-mitochondrial contacts and Unfolded Protein Response in Friedreich’s Ataxia iPSC-derived neurons**

Priyanka Mishra^1, †^, Anusha Sivakumar^1, †^, Avalon Johnson^2^,^3^, Carla Pernaci^2^,^3^, Anna S. Warden^2,3^, Lilas El-Hachem^1^, Emily Hansen^2,3^, Rafael A. Badell-Grau^1^, Veenita Khare^1^, Gabriela Ramirez^2,3^, Sydney Gillette^2,3^, Angelyn B. Solis^1^, Peng Guo^4^, Nicole Coufal^2,3,#^, Stephanie Cherqui^1,#^

**Supplementary Figures**

**Supplementary Figure S1.** **Frataxin gene and protein expression profile across the different neuronal cell lines.** ddPCR quantification of *FXN* mRNA and western blot protein quantification of the FRDA compared to their isogenic control or editing using our CRISPR/Cas9 approach. Data are represented as Mean + SEM and analyzed with Student’s *t* test where **p< 0.01and ns is not significant.

**Supplementary Figure S2. Apoptosis in FRDA neurons carrying longer GAA repeats. (A)**1 week and **(B)** 2 weeks differentiated 850 FRDA neurons immunolabelled for ß-tubulin III and Caspase-3.

**Supplementary Figure S3. Gene editing of the FRDA GAA intronic repeat.** (A) Principal component analysis of the transcriptomic samples from FRDA neurons, isogenic gene repeat corrected controls and gene edited samples. (B) Heat map of DEGs.

**Supplementary Table S1.** Primer sequences for qPCR studies.

**Data availability**

The RNA sequencing datasets from FRDA, edited, and isogenic control for this study can be found in the Gene Expression Omnibus (GEO). The accession number is GSE244886.
